# Do We Have the Right to Challenge the Rules of Nature Using Science and Technology Tools?

**Published:** 2019

**Authors:** Mohammad Reza Sadeghi

This month, newspapers and websites released the news referring to the birth of twin girls by the world's oldest mother. A 74-year-old Indian woman and an 82-year-old Indian man, who have been married for 57 years were treated after 30 years of postmenopause. She became pregnant using donated eggs at an infertility clinic in Guntur, India. This treatment resulted in delivery of twin girls (1).

This news disseminated confusion and received blame from medical policy makers, ethicists, lawyers and physicians, that why third-party infertility treatment should be ordered to such aged couple and debates about the future of children. Interestingly, and perhaps unfortunately, the report states that one day after the birth of these babies, her husband had heart attack, admitted to intensive care unit, besides the mother who was at special care in this unit due to the complications of pregnancy and twin delivery.

In addition, numerous extremely old cases have been reported to give birth to live babies previously; for example, in 2008, a pregnant woman, named Omkari Panwar, in India aged 70 or 72 years had live birth. However, her age was not confirmed due to the lack of a formal birth certificate. In 2016, another Indian woman, Daljinder Kaur, delivered a boy at the age of 72 (1). According to literature, an egg donation pregnancy resulted in multiple gestations at age 50. Immediately following delivery, hypertension was observed with nausea and vomiting. A few hours later, the case was diagnosed with HELLP syndrome and died of cerebral hemorrhage (2). Similar to the above case, in most of these cases, the mothers died shortly after delivery due to pregnancy and delivery complications or even other illnesses such as cancer.

Nature has restricted the life span of a woman's reproduction, but advances in science and technology can improve chances of getting pregnant through hormone replacement therapy and eggs or embryos donation, or even the preservation of the eggs or embryos of mothers at younger age. Furthermore, human pregnancy beyond the biological barriers is unnatural. The fact that some grandparents successfully take care of their grandchildren is not essentially sufficient to justify the use of reproductive technologies for pregnancy in post-menopausal women after 50 (3).

In fact, these grandparents, who are now parents of children, are unable to supply the psychological, economic, and physical demands of these children and maintain long-term parenting relationships. In addition, in most cases reported these children lost one or both parents before they reached adolescence which is the most stressful event of life that is beyond the endurance of children and adolescents. In addition, another problem with these pregnancies is the advanced paternal age, which is associated with increased birth anomalies and congenital disabilities due to genetic defects, as well as an increased risk of autism and schizophrenia (3).

Therefore, egg and embryo donations should be discouraged for older women who are also suffering from medical problems by providing extensive counseling that these risks are exacerbated by pregnancy and childbirth. Since the large numbers of medical problems are pregnancy-related, especially following multiple pregnancies, elective single embryo transfer (eSET) should be an unchanging rule for all ART services for pregnancy at advanced age.

The menopausal practitioner and obstetricians should provide more realistic insights about the evidence-based facts of maternal, fetal and neonate risks in pregnancy at advanced age. Clinicians should also advise the couples that you are at risk of not having a baby whenever you decide to delay your pregnancy and childbirth.

There is no comprehensive guideline for maternal health of over aged candidates for pregnancy, although the American Society for Reproductive Medicine (ASRM) has stated that healthy women over 50 who have conceived through egg donation are at high gestational risks. Therefore, it is recommended that over aged women be prohibited from receiving this service (4).

Although we recognize the obvious complications of pregnancy and childbirth of very old mothers as well as some of complications in their infants, we have no knowledge or prognosis of the long-term health consequences of these children at adulthood and geriatric era.

Finally, in the creation of universe, the restriction of women’s reproductive period provided wisdom and rational reasons, but we have come to the war of nature with the tools of science and technology. However, we must consider the fact that in similar cases, such as damaging the nature and environment ecology, we have witnessed the consequences of such activities for a long time which make us to contemplate and regret for all previous deed. Thus we need to respect the rules of nature and limit our challenges.
